# Current status of the remaining Mexican cloud forests: landscape findings and conservation initiatives

**DOI:** 10.7717/peerj.18386

**Published:** 2024-10-24

**Authors:** Wesley Dáttilo, Sergio A. Cabrera-Cruz, César A. Gallo-Gómez, Juan Carlos Serio-Silva, Rafael Villegas-Patraca

**Affiliations:** 1 Red de Ecoetología, Instituto de Ecología AC, Xalapa, Veracruz, Mexico; 2 Unidad de Servicios Profesionales Altamente Especializados, Instituto de Ecología AC, Xalapa, Veracruz, Mexico; 3 Red de Biología y Conservación de Vertebrados, Instituto de Ecología AC, Xalapa, Veracruz, Mexico

**Keywords:** Biological conservation, Effective mesh size, Landscape ecology, Mesophytic mountain forest, Shape complexity, Total edge

## Abstract

Tropical montane cloud forests are known for their unique biodiversity and their critical role in sustaining ecosystem services; however, approximately 50% of their original cover in Mexico was estimated to have been lost by 1998. The Mexican ecoregion that supports these ecosystems experienced one of the highest rates of deforestation between 2001 and 2021. Thus, a more recent evaluation of Mexico’s cloud forests is required. There is limited data on the landscape structure of cloud forests in Mexico, despite the possible application of landscape factors in conservation planning. Here, we estimated the average total area, number of patches, effective mesh size, total edge, and the shape of mixed forests that was present in 2020 within polygons of cloud forests defined in 1999 by Mexico’s National Commission for the Use and Knowledge of Biodiversity (CONABIO for its acronym in Spanish). We estimated land cover using data from the North American Land Change Monitoring System, which classifies cloud forests as mixed forests. We found that eight out of the 109 polygons have no mixed forests and that an average of 49% of the 1,768,914 ha of cloud forests polygons are now covered by mixed forests distributed across 13 states. Additionally, within the remaining 101 polygons that do contain this type of vegetation, mixed forest is distributed on average across 140 patches (range = 1–1,473); 80% of these forests have very low effective mesh size values; 90% of them have low total edge values (<2,000 km); and their shapes tend to be uniformly distributed. Furthermore, most of cloud forest polygons are located outside of federal protected areas. Overall, our results suggest that the remaining Mexican cloud forests are extremely vulnerable and fragmented and that their extent has steadily declined since 1999. To ensure the survival of Mexican cloud forests, it will be crucial to prioritize high-diversity areas, strengthen protection in critical zones, establish ecological corridors, encourage sustainable practices, and actively engage local communities. This study highlights the complex issues and inherent heterogeneity that characterize cloud forest ecosystems in Mexico and provides crucial insights for conservation.

## Introduction

The rapid alteration of natural habitats poses a significant threat to biodiversity and the essential ecosystem services required for Earth’s life (Poschlod, Bakker & Kahmen, 2005; Haines-Young, 2009). Expansion of the urban, agricultural, and industrial sectors has led to significant habitat losses, increased fragmentation, and detrimental effects on biological diversity across the board (Hewitt et al., 2010; Tilman et al., 2017), including the emergence of infectious diseases in wildlife (Daszak, Cunningham & Hyatt, 2000). Human development’s fragmentation of ecosystems increases barriers to animal movement, posing a greater risk to wildlife and increasing the likelihood of species disappearing (Van Dyck & Baguette, 2005; Rocha et al., 2021). Land-use changes not only impact biodiversity but also threaten essential ecosystem services like nutrient cycling, soil fertility, pollination, and water purification for human life (Hasan et al., 2020). These impacts are particularly concerning in the context of tropical forests due to their significant global biodiversity and climate regulation (Stork et al., 2009; Wright, 2010). Therefore, the alteration of habitat use irrevocably undermines complex and interdependent ecosystems in these crucial areas.

Tropical montane cloud forests, referred to as “cloud forests” hereafter, are renowned as some of the planet’s most diverse ecosystems due to the significant presence of endemic and endangered species (Myers et al., 2000). Forests at intermediate elevations provide moist and cool conditions for rich biodiversity adapted to misty environments, thanks to constant cloud cover (Hamilton, Juvik & Scatena, 1995; Rehm, 2014). Cloud forests significantly contribute to hydrological cycles by supplying water to nearby areas and influencing regional climate through cloud interception, highlighting their importance for local and broader climate stability (Bruijnzeel, 2001; Bruijnzeel, Mulligan & Scatena, 2011a). Despite their biological importance, cloud forests face considerable vulnerability to climate change due to their specific climatic requirements and their limited and fragmented spatial distribution (Ponce-Reyes et al., 2012; Jiménez-García & Peterson, 2019; Los et al., 2021). This vulnerability is particularly pronounced in the Neotropics, where the decline in low-cloud cover is most significant compared to other biogeographic realms (Guzmán, Hamann & Sanchez-Azofeifa, 2024). Changes in regional temperature and precipitation patterns are already affecting the extent and distribution of forests across various sites and ecological contexts (Pounds, Fogden & Campbell, 1999; Rehm & Feeley, 2015). Understanding and studying the current state of cloud forests is important for ecosystem conservation, planet stability, climate change mitigation, and for preserving valuable ecosystems for future generations.

In Mexico, cloud forests are predominantly located in mountainous regions, comprising less than 1% of the total land area (González-Espinoza et al., 2011; Luna-Vega et al., 2023). Nevertheless, they harbor the country’s richest biodiversity in terms of both plant and animal species (Rzedowski, 2006; González-Espinoza et al., 2011). The Mexican cloud forests represent the northernmost extension of this vegetation type in the New World (Luna-Vega & Magallón, 2010). These forests are best developed between 1,500 and 2,500 m above sea level and are characterized by a persistent and seasonal cloud layer (Rzedowski, 2006; González-Espinoza et al., 2011). Among the states hosting a larger extent of these ecosystems are Veracruz, Oaxaca, Puebla, Chiapas, and Hidalgo (Cayuela, Golicher & Rey-Benayas, 2006; González-Espinoza et al., 2011). Factors such as climate change, deforestation, habitat fragmentation, and atmospheric pollution are generating significant impacts on these forests in the country (Williams-Linera, Manson & Vera, 2002; Foster et al., 2003; Rojas-Soto, Sosa & Ornelas, 2012; Jiménez-García & Peterson, 2019). Additionally, human activities, including agricultural expansion and unsustainable logging, are contributing to the loss of cloud forest cover (Martínez et al., 2009; Ponce-Reyes et al., 2013; Luna-Vega et al., 2023). The impact of human activities led to a 50% reduction in the area originally covered by cloud forests in Mexico (Comisión Nacional para el Conocimiento y Uso de la Biodiversidad (CONABIO), 2010; Sánchez-Ramos & Dirzo, 2014).

It is imperative to implement effective conservation measures for cloud forests in Mexico, such as creating protected areas, implementing ecosystem restoration programs, and promoting sustainable practices, to ensure the long-term preservation of these valuable ecosystems (Toledo-Aceves et al., 2011; Rojas-Soto, Sosa & Ornelas, 2012; Jiménez-García & Peterson, 2019). Accurate documentation of the current state of cloud forests allows for the identification of critical areas that require immediate intervention, whether through the establishment of natural reserves, the application of sustainable forest management practices, or the education and involvement of local communities in conservation programs. However, despite the potential use of landscape parameters in conservation planning (Ribeiro et al., 2009), information on landscape structure in Mexican cloud forests is only available for small regions. An updated, broader-scale understanding of the status of cloud forests in the country will allow making informed decisions to be made and strategies developed that are tailored to local conditions, thereby maximizing the effectiveness of conservation efforts. Preserving cloud forests is crucial for ensuring the survival of unique species, for maintaining vital services like water regulation, and for mitigating climate change.

Our present knowledge on the status of cloud forests in Mexico comes from the accumulation of localized efforts directed toward exploring individual patches of forest, or through the systematization of expert opinions and is not updated (*e.g*., Williams-Linera, 2002; Comisión Nacional para el Conocimiento y Uso de la Biodiversidad (CONABIO), 2010; Toledo-Aceves et al., 2011). The evaluation of land-cover vegetation of cloud forests in Mexico requires an updated approach, which can be achieved using remote sensing data products. For instance, the North American Land Change Monitoring System (NALCMS) provides land cover maps homogenized for all North America (Canada Centre for Remote Sensing et al., 2017), categorizing cloud forests within its mixed forests land-cover class (Comisión Nacional para el Conocimiento y Uso de la Biodiversidad (CONABIO), 2019). Remote sensing data and local knowledge can provide a comprehensive understanding of cloud forest distribution and the degree of threat in Mexico. These data can aid policymakers, conservationists, and researchers in informed decision-making and specific conservation measures to protect this vital ecosystem for future generations. In this study, we provide a comprehensive assessment of the current status of cloud forest in Mexico by estimating the average total area, number of patches, effective mesh size, total edge, and shape of NALCMS’s mixed forests within polygons of cloud forest delimited by Mexico’s National Commission for the Use and Knowledge of Biodiversity (CONABIO, for its name in Spanish). By assessing these key landscape metrics and analyzing the distribution patterns of mixed forests across states, we aim to contribute valuable insights that can inform conservation planning.

## Materials and Methods

### Cloud forest polygons and current land cover

To analyze the current status of cloud forests in Mexico, we initially obtained all polygons identifying the presence of cloud forests from a country-wide vector map originally produced by Mexico’s National Institute for Geography and Statistics (INEGI, for its name in Spanish) and modified/grouped by CONABIO (Comisión Nacional para el Conocimiento y Uso de la Biodiversidad (CONABIO), 1999). INEGI is responsible for, among other things, producing and updating land-cover maps for the country (https://www.inegi.org.mx/inegi/contenido/infogeo.html), while CONABIO promotes, coordinates, and supports efforts directed toward generating and increasing Mexico’s knowledge on its biological diversity (https://www.gob.mx/conabio/que-hacemos). Additionally, we downloaded Mexico’s 2020 land cover data in raster format from the NALCMS (Canada Centre for Remote Sensing et al., 2017). This land cover data is a collaboration between the governments of Mexico, the United States of America, and Canada to promote the environmental cooperation between those parties and is operated by the Commission for Environmental Cooperation (http://www.cec.org/). The raster downloaded categorizes land cover in 19 classes with a 30-m spatial resolution based on Landsat satellite imagery (Commission for Environmental Cooperation (CEC), 2023). These classifications are harmonized across borders derived from the combination of geospatial data from the three countries. We used cloud forest polygons created in 1999 and land cover data from 2020 because there is some compatibility between their categories, and in this way get an idea of the change in forest cover between those years (see Data Analysis section).

The land-cover classification used by NALCMS is designed at three hierarchical levels: levels I and II are defined for the North American scale, and level III defines country-specific vegetation types (Latifovic et al., 2012). NALCMS Level 2 classification defines mixed forests as “Forests generally taller than three meters and more than 20 percent of total vegetation cover. Neither needleleaf nor broadleaf tree species occupy more than 75 percent of total tree cover but are co-dominant” (Commission for Environmental Cooperation (CEC), 2023). In NALCMS Level III classification for Mexico, mixed forests encompass cloud forests and oak-pine forests, along with their associated woody secondary vegetation (Comisión Nacional para el Conocimiento y Uso de la Biodiversidad (CONABIO), 2019). Mixed forests contain the species *Liquidambar styraciflua* (Altingiaceae), *Fagus grandifolia* (Fagaceae), *Chiranthodendron pentadactylon* (Malvaceae), *Engelhardtia americana* (Juglandaceae), *Carpinus tropicalis* (Betulaceae), and other genera such as *Clethra* (Clethraceae), *Ocotea* (Lauraceae), *Magnolia* (Magnoliaceae), *Inga* (Fabaceae), *Miconia* (Melastomataceae), *Quercus* (Fagaceae), *Podocarpus* (Podocarpaceae), *Oreopanax* (Araliaceae), *Ternstroemia* (Pentaphylacaceae), *Tilia* (Malvaceae), and *Prunus* (Rosaceae) (Llamas-Barba et al., 2015; Luna-Vega et al., 2023). Mexico participates in NALCMS through CONABIO and INEGI (Commission for Environmental Cooperation (CEC), 2023). Hence, all data products used for analysis have been vetted by the same institutions.

### Data analysis

We estimated the total area of all patches, the number of patches, the effective mesh size, the total edge, and the shape of mixed forest patches (Commission for Environmental Cooperation (CEC), 2023) within cloud forest polygons (Comisión Nacional para el Conocimiento y Uso de la Biodiversidad (CONABIO), 1999) using the functions *lsm_c_ca(), lsm_c_np(), lsm_c_mesh(), lsm_c_te()* and *lsm_c_shape_mn()* of the ‘landscapemetrics’ package (Hesselbarth et al., 2019, 2023) in R 4.3.1 (R Core Team, 2023). The total area is the area sum (in ha) of all patches of mixed forests within each polygon. The number of patches represents the number of patches of mixed forests within cloud forest polygons. The effective mesh size is an “aggregation metric” related to patch structure representing the degree of fragmentation of a landscape (in ha), with smaller values indicating higher fragmentation. The total edge is the total length (in km) of the boundaries between adjacent patches of different land cover types and represents a combination of fragmentation and heterogeneity within each cloud forest polygon. Shape describes the proportion of patch edge relative to patch sizes within each cloud forest polygon, with greater values of this index indicating fragments with more complex shape, mainly because most of the habitat they possess is exposed to edge conditions.

We estimated the Euclidian distance from each cloud forest polygon (Comisión Nacional para el Conocimiento y Uso de la Biodiversidad (CONABIO), 1999) to the closest Natural Protected Area (NPA) based on the map of the Mexican National System of NPAs (Comisión Nacional de Áreas Naturales Protegidas (CONANP), 2023) using the *st_distance()* function of the R package ‘sf: Simple Features for R’ (Pebesma, 2018; Pebesma et al., 2023; Pebesma & Bivand, 2023). We identified whether cloud forest polygons were partially or fully within NPAs using the ‘*sf*’ function *st_filter()* with the *st_overlaps()* and *st_covered_by()* predicates, respectively (Pebesma et al., 2023). We further estimated, for each cloud forest polygon, the area within NPAs using a combination of the functions *st_intersection()* and *st_area()* (Pebesma et al., 2023).

## Results

**Figure 1:**
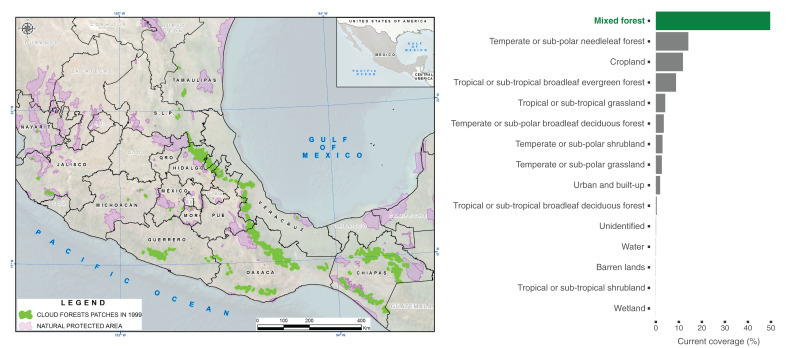
Distribution of cloud forests in 1999 and current land cover types within polygons. Left: Distribution of polygons identifying the presence cloud forests in Mexico (in green) from a country-wide vector map (Comisión Nacional para el Conocimiento y Uso de la Biodiversidad (CONABIO), 1999), highlighting terrestrial Natural Protected Areas in the country (in purple). Right: Average proportion of mixed forests and other current land cover types within the original Cloud Forest polygons, according to the Commission for Environmental Cooperation (Commission for Environmental Cooperation (CEC), 2023).

The approximately 886,486 ha of mixed forests that we identified in Mexico within CONABIOS’s cloud forest polygons spread across 13 different states: Chiapas, Guerrero, Hidalgo, Jalisco, Mexico, Michoacán, Morelos, Nayarit, Oaxaca, Puebla, San Luis Potosí, Tamaulipas, and Veracruz (but see Instituto Nacional de Estadística y Geografía (INEGI), 2021; Luna-Vega et al., 2023). It is noteworthy that a small portion of the cloud forest, primarily from the state of Hidalgo, extends into Querétaro (Cartujano et al., 2002). We found that eight of the 109 polygons originally proposed by CONABIO for cloud forests did not contain mixed forests. Furthermore, only 49% of the 1,768,914 ha of land that CONABIO suggested contained cloud forests in 1999 corresponded with mixed forests as estimated by the NALCMS in 2020. The surface that CONABIO suggested presents cloud forests also contained temperate or sub-polar needleleaf forest (14.12%) and cropland (11.78%) and 14 other land cover types (Fig. 1).

**Figure 2:**
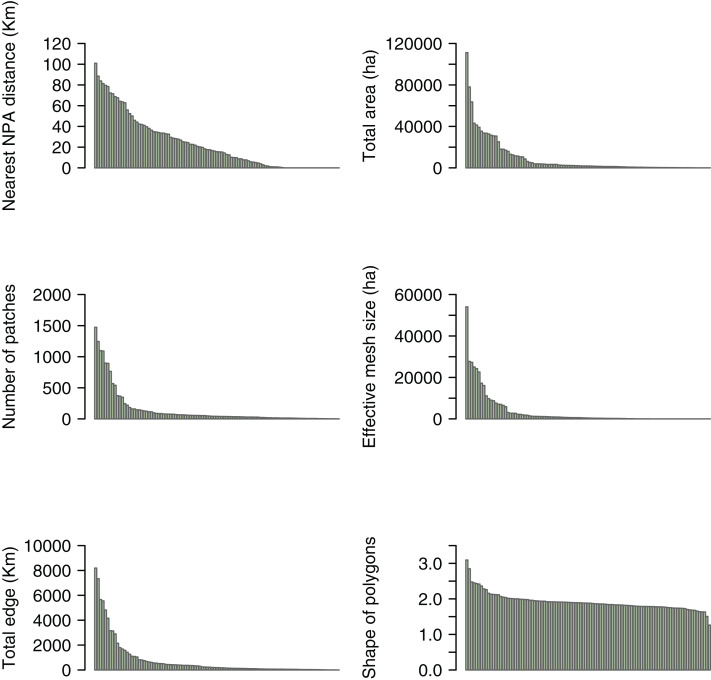
Landscape descriptors within CONABIOS’s Cloud Forest polygons in Mexico. Distribution of distances from Cloud Forest polygons to the closest Natural Protected Areas (NPAs), as well as total patch area (ha), number of patches, effective mesh size (ha), total edge (Km), and shape of mixed forest within CONABIOS’s Cloud Forest polygons in Mexico.

We found an average of 140 ± 278 patches of mixed forests (Mean ± SD, Range = 1–1,473) within the polygons, and the amount of mixed forest within most of these polygons (60%) was <2,500 ha (Mean ± SD = 8,777 ± 17,395 ha, Range = 0.9–111,153 ha) (Fig. 2). Taken together, these results suggest that most of the remaining cloud forest in Mexico is highly fragmented and spread in relatively small patches. Additionally, we discovered that 83% of the patches of mixed forests exhibit very low effective mesh size values (<3,107 ha), indicating a high degree of fragmentation. In fact, most mixed forest patches had low total edge values (90% have <2,000 km, *i.e*., small patches). We also found that the shape of the remaining patches tends to be distributed uniformly, indicating significant similarity in the proportion of patch edge relative to patch sizes.

**Figure 3:**
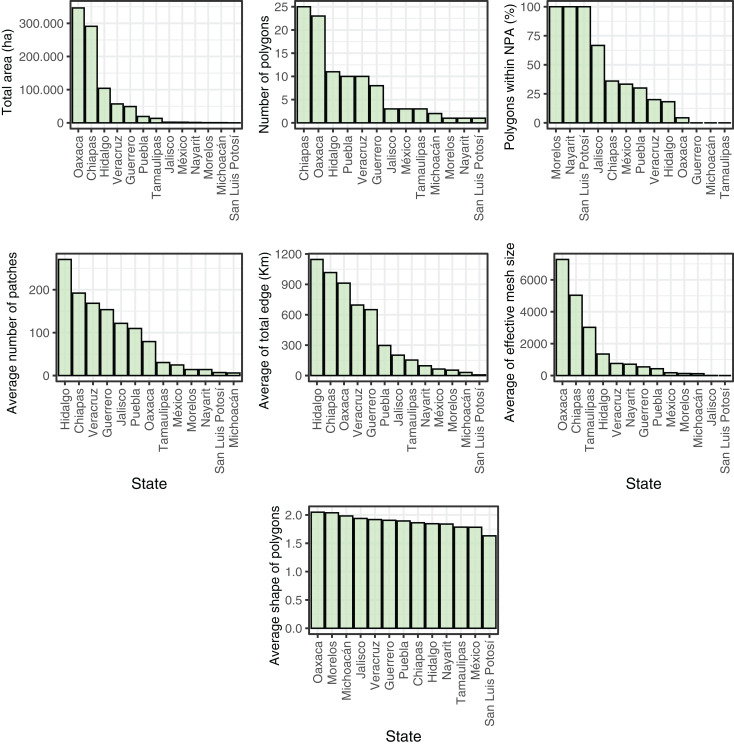
Landscape metrics of mixed forest patches in the States of Mexico. Distribution of the total patch area (ha), number of polygons, polygons within Natural Protected Areas (NPAs), average number of patches, average of total edge (Km), average of effective mesh size (ha), and average shape of mixed forest patches in the States of Mexico.

We observed that 77.2% of CONABIOS’s polygons (*n* = 78) are located outside NPAs, with only 17.8% (*n* = 18) being within or adjacent to an NPA. Among those outside, ~20% are at a distance greater than 50 km (29.8 ± 25.07 km). Regarding the states, we found that Oaxaca and Chiapas have the largest total area of mixed forest within CONABIOS’s cloud forest polygons, with 346,384 ha and 291,028 ha, respectively. In fact, the two largest patches of mixed forests are precisely in Oaxaca and Chiapas, covering 111,153 and 78,028 ha, respectively, while the smallest ones occur in Hidalgo (0.9 ha) and Veracruz (0.99 ha). Chiapas and Oaxaca had the highest number of mixed forest patches, with *n* = 25 and *n* = 23, respectively, while Morelos, Nayarit and San Luis Potosí had the fewest patches (*n* = 1). Morelos, Nayarit, and San Luis Potosí were the only states where all their polygons (*n* = 1) were located within NPAs. In contrast, Guerrero, Michoacán, and Tamaulipas had all their cloud forest polygons situated outside of NPAs (but see Discussion). Jalisco, Chiapas, México, Puebla, Veracruz, Hidalgo, and Oaxaca had only part of their polygons located within NPAs. Hidalgo had the highest average number of patches within the polygons (*n* = 270), while San Luis Potosí (*n* = 7) and Michoacán (*n* = 6) had the lowest averages. Hidalgo also had the highest average total edge (*n* = 1,145 km), and San Luis Potosí had the lowest (*n* = 6.81 km). Furthermore, we found that Oaxaca and Chiapas had lower average degrees of fragmentation in their mixed forest remnants (*i.e*., high values of effective mesh size), while the states of Jalisco and San Luis Potosí had very low effective mesh size values on average (*i.e*., a high degree of fragmentation in their remnants). Finally, we observed that the shape of the patches was uniformly distributed among the states (Fig. 3).

## Discussion

In this study, we conducted a quantitative assessment of the current status of cloud forests in Mexico from a landscape perspective. Our observations reveal a diverse mosaic of land cover types in 2020 within the cloud forest polygons proposed by CONABIO in 1999. Notably, most of these polygons are outside of federal protected areas and exhibit a high degree of fragmentation. These results highlight the threat faced by this ecosystem in Mexico, mainly due to a combination of natural factors (*e.g*., episodic/catastrophic ecological events such as hurricanes) as well as human-induced pressures (*e.g*., climate change, land-use change, and species introductions) (Ponce-Reyes et al., 2012; Falcão et al., 2022; Luna-Vega et al., 2023).

Previous studies have shown that cloud forests have undergone (and continue to undergo) a high degree of environmental disturbance, including reductions in low-cloud cover (Lawton et al., 2001; Martínez et al., 2009; Toledo-Aceves et al., 2011). This disturbance has led to widespread landscape modifications globally (Hamilton, Juvik & Scatena, 1995; Sánchez-Ramos & Dirzo, 2014), particularly within the Neotropics and Indomalayan realms (Guzmán, Hamann & Sanchez-Azofeifa, 2024). Here, we identified that within cloud forest polygons from 1999 in Mexico, a mosaic of land cover types currently exists. Temperate or sub-polar needleleaf forests, cropland, tropical or sub-tropical broadleaf evergreen forests, and tropical or sub-tropical grassland account for >38% of the composition within these polygons while mixed forests, which contain cloud forests (Comisión Nacional para el Conocimiento y Uso de la Biodiversidad (CONABIO), 2019), cover <50% of the polygons. Despite this heterogeneity, the remaining cloud forests still allow the coexistence of a rich biodiversity and provides important ecosystem services, such as climate regulation, soil nutrient cycles, and water supply (Bruijnzeel, 2004; Bruijnzeel, Scatena & Hamilton, 2011b; González-Espinoza et al., 2011). Therefore, these factors should be considered in ecology and conservation studies, as well as in decision-making processes. Ignoring this high variability could lead to decisions that compromise the ability of these ecosystems to sustain themselves and impact their resilience to environmental changes, jeopardizing the health of the ecosystems and their benefits for future generations. Consequently, recognizing and accounting for the intricate dynamics and ecological services provided by the diverse land cover types within cloud forests is crucial for effective ecological and conservation studies and for making informed decisions to ensure the long-term health and resilience of these ecosystems.

While the nomenclature of land cover types assigned in the data products used for analysis may seem disparate (cloud forests and mixed forests), both institutions in Mexico responsible for the creation of the cloud forest polygons for the country (Comisión Nacional para el Conocimiento y Uso de la Biodiversidad (CONABIO), 2010) are part of the consortium responsible for the land cover dataset (Canada Centre for Remote Sensing et al., 2017; Commission for Environmental Cooperation (CEC), 2023). Furthermore, one such organization (CONABIO) vetted the categorization of cloud forests within the mixed forest class used by NALCMS (Comisión Nacional para el Conocimiento y Uso de la Biodiversidad (CONABIO), 2019). Consequently, we consider that the extent of mixed forests in the NALCMS data product is indicative of the extent of cloud forests within CONABIO’s polygons. Moreover, the proportion of the three most abundant NALCMS forest types within cloud forest polygons (Fig. 3) adds up to <80% of the polygons area. This indicates that even if the NALCMS data product had misclassified cloud forests as some other land cover class instead of mixed forests, it would still detect coverage of treed vegetation lower than purported by the polygons. INEGI updates its country-wide land cover maps for Mexico every 3 to 5 years. We used their data product aggregated by CONABIO for 1999 to put our results in the context of a benchmark indicating that the original cover of cloud forests in Mexico around that year had decreased by 50% (Challenger et al., 1998). A similar result was obtained by a later work that analyzed the extent of cloud forests in Mexico by comparing the area covered by polygons proposed by INEGI for different years (Sánchez-Ramos & Dirzo, 2014). In that study, authors proposed that the amount of cloud forests in Mexico by 2007 was 1,697,600 ha (16,976 km²). This number is similar to the total area covered by the polygons used here (1,768,914 ha). Using NALCMS land cover data from 2020, we estimate only 881,071 ha of mixed forests within said polygons. Since the NALCMS mixed forest class for Mexico also contains oak-pine forests (Comisión Nacional para el Conocimiento y Uso de la Biodiversidad (CONABIO), 2019), we might be overestimating the amount of cloud forest within the polygons. While our estimation is drastically lower than previous ones (Sánchez-Ramos & Dirzo, 2014), it comes after a period in which the Mexican transition zone (Morrone, 2020), which includes the main mountain chains in Mexico and harbors cloud forests in the country, suffered a deforestation rate of 32,840 ha/year between 2001 and 2018 (Comisión Nacional Forestal (CONAFOR), 2020). At the very least, our results suggest the urgent need for a dedicated effort to estimate the amount of cloud forests remaining in Mexico with higher precision. Indeed, Helmer et al. (2019) found that the Trans-Mexican Volcanic Belt, Sierra Madre Oriental, and Veracruz exhibit the lowest cloud forest cover, with reductions of up to 82% due to significant deforestation.

We also emphasize the high degree of vulnerability of Mexican cloud forests as consequence of land-use change (Williams-Linera, 2002; Sánchez-Ramos & Dirzo, 2014; Jiménez-García & Peterson, 2019; López-Arce et al., 2019). This is due to many polygons being located outside NPAs, with a significant portion situated far from these areas (Toledo-Aceves et al., 2011). Consequently, cloud forests face constant exposure to various threats, including deforestation, habitat fragmentation, agricultural/territorial pressures, climate change, and biological invasions (Luna et al., 2006; Luna-Vega et al., 2023), posing risks to biodiversity, ecosystem services, and the resilience of these areas to environmental changes. Additionally, Ponce-Reyes et al. (2012) suggested that over 90% of cloud forest within currently federal protected areas in Mexico will not be climatically suitable for this ecosystem by the year 2080. Changes in regional temperature and precipitation patterns are considered key factors shaping the extent and distribution of this type of forest (Still, Foster & Schneider, 1999). While protected areas globally have mitigated some of the decline in cloud forest cover, a significant proportion of loss in tropical cloud forests continues despite formal protection (Karger et al., 2021). Moreover, Helmer et al. (2019) highlight that six Mexican ecoregions contain substantial areas of unprotected, intact forest within regions least affected by climate change. Thus, it underscores the importance of proposing new strategies, such as expanding the number and size of protected areas with better climatic conditions to safeguard Mexican cloud forests.

Furthermore, we observed that most of the mixed forests within the polygons have a small extension (lower than 2,500 ha) and exhibit a high degree of fragmentation (low effective mesh size values). These small fragments constitute a large fraction of the remnants, which is crucial for mitigating threats and playing a fundamental role in maintaining biodiversity and providing ecosystem services (Ribeiro et al., 2009; Liu & Slik, 2014; Amaya-Espinel & Hostetler, 2019). Therefore, the implementation of conservation policies and sustainable practices for small fragments should not be neglected (Riva & Fahrig, 2022, 2023). On the other hand, we also found that the shape of the remaining patches tends to be uniformly distributed among them. Such uniformity in the shape of habitat fragments could compromise the ecosystem’s ability to sustain a wide variety of species and to respond to environmental challenges. Uniform patches would be exposed to the same edge effects, not offering a diverse range of microenvironments and resources, limiting the vagility of some species and the interconnection between different ecosystems (Turner, Gardner & O’Neill, 2001; Yamaura et al., 2008). This is extremely relevant because fragment shape appears to be an important driver of bird species richness in Mexican cloud forests (Martínez-Morales, 2005). Therefore, when planning and implementing conservation measures for Mexican cloud forests, it is of utmost importance to consider the diversity of habitat shapes and types to promote more resilient and biodiverse ecosystems (Hill & Curran, 2003; Pardini et al., 2010; Liu & Slik, 2014).

When analyzing the current state of cloud forests within the country, we observed that, despite the uniformity in the shape of patches across states, the current situation of other parameters is highly asymmetric among Mexican states. For instance, we found that the states of Oaxaca and Chiapas have the largest average of mixed forest within the polygons, the highest number of mixed forest patches, and a higher degree of fragmentation. On the other hand, the state of Morelos, Nayarit, and San Luis Potosí has all polygons within protected areas, while other states have all polygons outside (Guerrero, Michoacán, and Tamaulipas), showing considerable inadequacy of the present federal NPA system for Mexican cloud forests (Jiménez-García & Peterson, 2019). It should be noted, however, that some cloud forests might be protected by other mechanisms. For example, the El Cielo Biosphere Reserve in Tamaulipas is a state-level protected area that operates independently from the NPA system and where cloud forests span approximately 160 km² (González-Medrano, 2005). Moreover, it is important to note that Morelos was the state where the current situation of cloud forests showed the least amount of edge and number of polygons but a high degree of fragmentation.

In the context of alternative conservation mechanisms, the Areas Voluntarily Designated for Conservation or AVDCs (*Áreas Destinadas Voluntariamente para la Conservación*, in Spanish), represent conservation areas protected by local communities. AVDCs operate independently of the NPA federal system, as the state-level protected areas do, and are efficient in conservation terms (Martin et al., 2011; Briones-Salas et al., 2016; Contreras-Medina et al., 2022). Hence, for example, while the state of Oaxaca has fewer NPAs than other states (Contreras-Medina et al., 2022) thus suggesting a lower representation of cloud forests in protected areas (see Ponce-Reyes et al., 2013; Fig. 3), ADVCs are distributed across the state, aiding in the protection of cloud forests.

Given the observed asymmetry and heterogeneity in the current situation of cloud forests among Mexican states, various conservation approaches can be considered. These approaches include the following: (i) prioritizing areas with higher diversity and lower fragmentation; (ii) strengthening protection in critical conservation areas to enable effective implementation and enforcement of environmental regulations; (iii) establishing ecological corridors in regions with high fragmentation to facilitate species movement; (iv) promoting sustainable practices in areas where human pressure is high; (v) constantly monitoring biodiversity to provide scientific foundations that help adjust conservation strategies based on new information and changes in environmental conditions; (vi) involving local communities in the implementation of more effective conservation practices, ensuring that adopted approaches align with local needs and promote sustainable development; (vii) implementing restoration initiatives in degraded areas to enhance ecosystem resilience and biodiversity, thereby complementing conservation efforts by regenerating habitats and enhancing the overall functionality of cloud forest ecosystems; and (viii) fostering collaboration between government agencies, non-governmental organizations, and local communities to maximize resources and efforts directed toward conservation. By integrating protective measures in vital areas, advocating sustainable practices, and engaging local communities proactively, comprehensive approaches can be developed to tackle various conservation needs throughout the country (Comisión Nacional para el Conocimiento y Uso de la Biodiversidad (CONABIO), 2010).

### Methodological limitations of the study

One of the primary limitations of our study stems from the analysis of the land cover classes categorized by NALCMS as mixed forest, which may not fully capture the spatial distribution of cloud forests as defined by Comisión Nacional para el Conocimiento y Uso de la Biodiversidad (CONABIO) (1999). Our analysis revealed instances where mixed forests were situated outside designated cloud forest areas, indicating a lack of spatial coincidence. Consequently, this discrepancy may lead to an underestimation of the total number of polygons representing cloud forests. Moreover, the NALCMS data utilized in our analysis do not explicitly identify cloud forests as a distinct vegetation category; rather, they encompass both cloud forests and oak-pine forests, as well as their associated secondary woody vegetation. Therefore, mixed forest areas not only encompass potential cloud forest locations but also incorporate other vegetation types, thereby influencing the outcomes of our analyses. Even with these limitations, our results offer insightful information.

## Conclusions

While previous global studies on cloud forests offer broad insights about different aspects of this ecosystem, our work focuses specifically on Mexico and expands the existing literature with detailed insights that are crucial for local conservation efforts and policymaking. In general, we found that the current state of cloud forests in Mexico suggests a harsh reality, as recently proposed by Ochoa-Ochoa, Mejía-Domínguez & Bezaury-Creel (2021). By 2020, cloud forests in Mexico seem to have lost nearly 50% of their coverage remaining in 1999, in addition to the 50% that had already been lost by then (Comisión Nacional para el Conocimiento y Uso de la Biodiversidad (CONABIO), 2010; Challenger et al., 1998). Moreover, more than half of Mexico’s current cloud forest could disappear by 2080 solely due to climate change (Ponce-Reyes et al., 2012). Our analysis unveils a mosaic of different land cover types within areas that in 1999 were considered to contain continuous cloud forests, as well as a high fragmentation, demonstrating that these ecosystems currently face a complex and heterogeneous situation that should be considered when planning their conservation for biodiversity maintenance and ecosystem service provision. We also underscore the significant vulnerability of Mexican cloud forests, reflected in their low representativeness within NPAs (Ponce-Reyes et al., 2012; Jiménez-García & Peterson, 2019), thus warranting urgent conservation measures. The observed asymmetry among states, with varying levels of protection and degrees of fragmentation, reinforces the need for adaptive approaches tailored to the specific needs of each region. Nevertheless, an analysis of the representativeness of cloud forests considering all types of protected areas in Mexico (AVDCs, and NPAs at federal, state and municipality level), is needed.

The involvement of local communities and collaboration between governmental entities and non-governmental organizations will be crucial for the successful implementation of conservation strategies. Our results underscore the urgency of implementing effective conservation measures, such as creating protected areas tailored to the protection of the remaining cloud forests and addressing fragmentation issues, to ensure the long-term preservation of these ecologically significant cloud forests. In summary, our study highlights the continued loss of cloud forests in Mexico. We emphasize the need for integrative approaches (*e.g*., ground-truthing) that consider the heterogeneity and asymmetry present in different states of the country. The effective implementation of such strategies is vital to ensure the preservation of these unique ecosystems and to provide lasting benefits for future generations.

## Supplemental Information

10.7717/peerj.18386/supp-1 Supplemental Information 1 Values of the landscape descriptors within each Cloud Forest polygon. Total area of all patches, number of patches, effective mesh size, total edge, and shape of Mixed forest patches within cloud forest polygons using the functions *lsm_c_ca(), lsm_c_np(), lsm_c_mesh(), lsm_c_te()* and *lsm_c_shape_mn()* of the ‘landscapemetrics’ package in R 4.3.1

## Additional Information and Declarations

### Data Availability

The following information was supplied regarding data availability: The raw data are available in the Supplemental File.
